# Partition Function Zeros and Heat Capacity Decomposition Reveal HP Protein Foldability

**DOI:** 10.3390/polym17212956

**Published:** 2025-11-06

**Authors:** Sing-Shuo Huang, Chi-Ning Chen

**Affiliations:** Department of Physics, National Dong-Hwa University, Hualien 97401, Taiwan

**Keywords:** protein folding, HP model, partition function zeros, heat capacity decomposition, kinetic Monte Carlo simulation

## Abstract

The heat capacity decomposition method, a well-established analytical approach in polymer thermodynamics for elucidating thermal transitions in homogeneous polymers, is extended here to heterogeneous systems. We demonstrate that the decomposition of heat capacity based on partition function zeros allows the identification of transition-like crossovers originating from compact low-energy states, thereby enabling the evaluation of the foldability of HP sequences. The occurrence of significant crossovers between the collapse and folding transitions indicates slow folding behavior, whereas their absence characterizes good folders. This criterion is further validated through kinetic Monte Carlo simulations of two representative sequences.

## 1. Introduction

Protein folding is fundamentally a thermodynamic process, at least for small protein [1]. The unique native state is the minimum free energy state. How a protein is able to locate its native state among an astronomical number of excited conformations so rapidly remains a central puzzle [2]. A widely accepted explanation is that evolved proteins fold on a free-energy funnel landscape [3,4]. In practice, however, depicting the funnel is challenging due to the complexity of the vast, high-dimensional conformational space. The theoretical study of protein folding has a long history [5,6,7,8,9]. More recently, AlphaFold [10,11,12] has achieved a groundbreaking breakthrough in protein structure prediction. It can predict the three-dimensional structure of a given amino acid sequence with several hundred residues in minutes or hours—tasks that previously could take years. The unexpected success of AlphaFold relies on innovations in neural network architectures, accelerated by advances in computer hardware, and the availability of extensive sequence and structure data in protein databases. It is essentially a computational, knowledge-based approach, which differs significantly from previous theoretical methods. While AlphaFold has achieved impressive accuracy in structure prediction, a fundamental theory of protein folding based on basic physical principles still remains to be fully established.

In this paper, we utilize partition function zeros, a technique from statistical mechanics, to probe the foldability of protein sequences. The partition function is the central quantity in statistical mechanics, from which all thermodynamic functions can be derived. Partition function zeros are defined as the roots of the equation “partition function = 0”. These roots fully determine the properties of the partition function and are distribute in the complex temperature plane (for the canonical ensemble) or in the complex fugacity or magnetic field plane (for the grand canonical ensemble). Since the pioneering work of Yang and Lee [13,14], which related partition function zeros to singularities in phase transitions, steady theoretical development has been made in understanding the their behaviors. From the loci and density of partition function zeros, one can extract critical properties such as critical points, the order of phase transitions, and critical exponents [15,16,17]. However, when applied to systems with competing interactions—such as the heterogeneous HP model [18], in contrast to homogeneous interacting self-avoiding walks—the zeros typically form scattered patterns without clear loci [19,20]. To address this limitation, we developed the strategy of heat capacity decomposition [21], in which each partition function zero is associated with a corresponding heat capacity component, thereby providing the zero with a physical interpretation. This additional information enables the analysis of complex patterns of partition function zeros to become feasible. A brief introduction of heat capacity decomposition is provided in Section 2.

The HP model is the simplest coarse-grained model of protein folding. In this model, an HP chain is a self-avoiding walk composed of two types of amino acid beads. The 20 natural amino acids are classified as either hydrophobic (H) or polar (P), as hydrophobicity is considered the dominant driving force of protein folding [22,23]. The energy is defined as

(1) $$E=\sum_{<i,j>}\varepsilon_{A_{i}A_{j}}$$

where the amino acid types $A_{i}$ and $A_{j}$ can be either H (Hydrophobic) or P (Polar), and <i,j> means the *i*th amino acid and *j*th amino acid have nonconsecutive nearest-neighbor contacts. There are three types of contact energies, $\varepsilon_{HH},\;\varepsilon_{HP},\;\varepsilon_{PP}$, which satisfy the inequalities $\varepsilon_{HH}<\varepsilon_{HP}<\varepsilon_{PP}$ and $\varepsilon_{HH}+\varepsilon_{PP}<2\;\varepsilon_{HP}$. Here we adopt an integer-scaled version of the interaction parameters: $\varepsilon_{HH}=-10,\;\varepsilon_{HP}=-6,\;\varepsilon_{PP}=-3$, corresponding to the values −3.3,−2, and −1 used in [24]. With integer-valued energies, the partition function takes the form of a polynomial, which makes root-finding more tractable. We test other interaction parameters as well and find they does not lead to significant qualitative differences. The HP model, much like the Ising model, is related to many other problems. For example, determining the ground state of the HP model is an NP-complete optimization problem [25]. It also serves as a testing ground for numerous computational algorithms [26]. Constructing basic principles with such simplified models is often more efficient, and these principles are expected to remain valid for more realistic models as well.

After analyzing the folding properties of HP sequences using partition function zeros and heat capacity decomposition, we verified the results with Gillespie-type kinetic Monte Carlo simulations. Unlike the traditional Metropolis algorithm, this approach focuses on simulating real physical motion. Details of this method are provided in the next section.

## 2. Method

### 2.1. Heat Capacity Decomposition

With integer-valued contact energies, the partition function of an HP protein sequence with *N* amino acids can be expressed as a polynomial in the variable $x\equiv e^{\;1/(k_{B}T)}$:

(2) $$\begin{array}{cc} Z_{N}\left(x\right)= & \sum_{\mathrm{all}\;\mathrm{structures}}e^{-E/\left(k_{B}T\right)}=\sum_{\mathrm{all}\;\mathrm{structures}}{\left(e^{\;1/\left(k_{B}T\right)}\right)}^{\left(-E\right)}=\sum_{E=E_{0}}^{0}n\left(E\right)\;x^{\left(-E\right)}\\ = & n\left(E_{0}\right)\prod_{k=1}^{M}\left(x-x_{k}\right) \end{array}$$

where n(E) denotes the number of structures with energy *E*, *M* = $-E_{0}$ is the degree of the polynomial, and $x_{k}$ is the *k*-th zero of the partition function. The heat capacity can be obtained from the partition function as $C_{V}\left(x\right)=dU/dT$ and $U=-\partial /\partial \beta \;\ln Z_{N}$, where *U* is the internal energy and $\beta =1/(k_{B}T)$. After changes of variables,

(3) $$C_{V}\left(x\right)=x{\left(\ln\;x\right)}^{2}\frac{d}{dx}x\frac{d}{dx}\ln\;Z_{N}\left(x\right)=-x{\left(\ln\;x\right)}^{2}\sum_{k=1}^{M}\frac{x_{k}}{{\left(x-x_{k}\right)}^{2}}.$$

Since $Z_{N}\left(x\right)$ is a polynomial in *x*, its roots occur as complex conjugate pairs, with the exception of very few real roots located on the negative real axis. For each complex or real $x_{k}$,

xk(x−xk)2+xk*(x−xk*)2=2Re(xk(x−xk)2)=2(Re(xk)|xk|2−2|xk|2x+Re(xk)x2)(|xk|2−2Re(xk)x+x2)2.

Thus, we may define the heat capacity component as

(4) CV,k(x)≡−x(lnx)2Re(xk)|xk|2−2|xk|2x+Re(xk)x2(|xk|2−2Re(xk)x+x2)2

and

(5) $$C_{V}\left(x\right)=\sum_{k=1}^{M}C_{V,\;k}\left(x\right)$$

In this way, the heat capacity $C_{V}\left(x\right)$ is decomposed into individual components $C_{V,\;k}\left(x\right),1\leq k\leq M$, each of which is a real function of the real variable *x* and is contributed by the zero $x_{k}$.

In the thermodynamic limit, i.e., as the system size N→∞, the partition function zeros will touch the positive real axis if a phase transition occurs [13,14,27] and $C_{V}/N$ will diverge. For finite systems, the divergence manifests as a peak, and the peak position can reasonably be defined as the transition point. The roots closest to the real axis, refered to the first zeros, provide the leading contribution of $C_{V}$ due to ${\left(x-x_{1\mathrm{st}}\right)}^{2}$ term in the denominator, where $x_{1\mathrm{st}}$ denotes the first zero. Near the real part of $x_{1\mathrm{st}}$, the associated heat capacity component exhibits a pronounced peak, the position of which can also be used to define the transition point of the phase transition in small systems [15,28,29].

Similar to an interacting self-avoiding walk (SAW) [30], a protein-like HP chain undergoes a collapse transition, the most common phase transition in polymers, moving from coil to globule states. A second transition, the folding transition from the globule to the native state, is inherently dependent on the specific amino acid sequence. Using the peak positions of the corresponding heat capacity components, the collapse temperature $T_{\theta }$ and the folding temperature $T_{f}$ can be defined. Both transitions contribute to the heat capacity, causing the shape of $C_{V}$ to vary markedly across different sequences. The $C_{V}$ peaks of different transitions often mask one another. Through heat capacity decomposition, the overlapping contributions of the two transitions can be separated, providing a clearer view of their respective roles. This heat capacity decomposition technique is specifically designed for small systems that undergo multiple phase transitions, typically indicated by multiple or irregularly shaped heat capacity peaks. Additional details on the method can be found in [21].

### 2.2. Kinetic Monte Carlo Simulation

We employ the kinetic Monte Carlo simulation based on the Gillespie algorithm [31,32] to detect the folability of HP sequences. The principles and procedures of this method are briefly described below. Suppose there are *K* possible moves for a HP chain. The transition probability per unit time for each move *i* is $W_{i}$, with 1≤i≤K. Let $P_{i}\left(t\right)dt$ represent the probability that move *i* occurs at time *t*. This probability can be expressed as $P_{0}\left(t\right)\;W_{i}dt$, where $P_{0}\left(t\right)$ is the probability that no move occurs before *t*, and $W_{i}dt$ is the probability that the move *i* occurs within the interval dt. Deviding *t* into *n* equal parts, $P_{0}\left(t\right)$ can be writen approximately as ${\left(1-W_{T}\;t/n\right)}^{n}$, where $W_{T}=\sum_{i}W_{i}$. In the limit n→∞, $P_{0}\left(t\right)=e^{-W_{T}\;t}$. Thus, we get the final result:

(6) $$P_{i}\left(t\right)=\left(W_{T}\;e^{-W_{T}\;t}\right)\frac{W_{i}}{W_{T}}$$

In other words, the waiting time *t* for one move to occur follows an exponential distribution, and the probability that the move is type *i* is given by $W_{i}/W_{T}$. The value of *t* can be simulated using the standard transformation method:

(7) $$t=\frac{-\log\;\xi }{W_{T}}$$

where ξ is an uniformly distributed stochastic variable in the range [0,1]. It is worth noting that $W_{T}$ is updated after every move; hence, the exponential function and the probability ratio $W_{i}/W_{T}$ are not fixed. Howerver, ξ remains independent of these quantities, and <−logξ>=$\int_{0}^{1}-\log\;\xi \;d\xi =1$. Therefore, $<t>=<1/W_{T}>$, which is consistent with expectations.

The move probability $W_{i}$ here is taken to be the Metropolis type:

(8) $$W_{i}=\min\left(1,e^{-\Delta E_{i}/k_{B}T}\right)$$

i.e., for $\Delta E_{i}\leq 0$, $W_{i}=1$, and for $\Delta E_{i}>0$, $W_{i}=e^{-\Delta E_{i}/k_{B}T}$, where $\Delta E_{i}$ is the energy change associated with move *i*. When considering actual physical time, the rate $W_{i}$ must be multiplied by an additional constant, which represents the frequency of attempted moves for an amino acid. One step of the kinetic Monte Carlo simulation consists of selecting a move *i* from all possible moves according to the probability ratio $W_{i}/W_{T}$, and advancing the simulation time by $-\log\;\xi /W_{T}$. After this move is performed, the list of possible moves needs to be updated. The procedure is then repeated to select subsequent move and update the system. Through successive moves, the system approaches thermal equilibrium at temperature *T*. We record the total time when the HP chain first reaches the lowest-energy state, i.e., the native state. This first-passage time is relevant to the kinetic accessibility of the native state at a constant temperature and can serve as a measure of the folding ability of a sequence [33].

## 3. Result

We consider all HP sequences with 16 HP amino acids on the simple cubic lattice. There are 2^16^ = 65,536 different HP sequences. Using exact enumeration [34], we compute the partition functions of all these sequences and filter out those without a unique ground state. This procedure leaves 5248 sequences as candidates for HP protein sequences. We then calculated the partition-function zeros of all these sequences and performed heat-capacity decomposition for several hundred of them. Table 1 presents 20 sequences randomly selected from the examined set. The table lists their ground-state energy $E_{0}$, $T_{f}$ and $T_{\theta }$. It can be observed that while $T_{\theta }$ varies in a relatively regular manner, $T_{f}$ exhibits considerable irregularity. This behavior closely resembles that found in the three-dimensional ISAW model [21,30], where the freezing transition temperature is irregular with respect to chain length because it strongly depends on the specific ground-state structures.

We can roughly classify the 20 sequences into two groups based on their heat capacity components between $T_{f}$ and $T_{\theta }$. The first group shows no significant heat capacity peaks in this range, while the second group does. This classification should be regarded as qualitative, since the significance of the peaks varies in a continuous manner. The appearance of these peaks in the second group suggests the existence of low-energy states that compete with the native state, forcing the native state to emerge only at lower temperatures and thus reducing $T_{f}$. Consequently, $T_{f}$ is generally higher in the first group and lower in the second. To further clarify these points, we choose two sequences in Table 1 as representatives of the two groups to present the results in details.

The two sequences are sequence No. 6 from the first group (referred to as sequence A) and sequence No. 18 from the second group (referred to as sequence B). They are not exceptional; similar conclusions would be obtained if other sequences were selected as examples. Sequence A can be regarded as a protein-like sequence that folds more rapidly, whereas Sequence B is a non-protein-like sequence that folds more slowly than Sequence A because it must pass through a crossover to reach the native state. Here, the term *crossover* refers to a smooth change in the physical state, not as abrupt as a phase transition, but still capable of producing a peak in the heat capacity. Finally, we will perform kinetic Monte Carlo simulations to verify these predictions.

The partition functions of sequences A and B are provided in the Appendix A. Following the convention described in [35], and considering both rotational and chiral symmetries, the degeneracies of the linear, planar, and three-dimensional structures are 1, 4, and 8, respectively. Consequently, the coefficient of the leading term of all 5248 partiton functions, $N(E_{0})$, is always equal to 8. The partition function zeros, i.e., the roots of the two polynomials, are solved with Mathematica.

**Figure 1:**
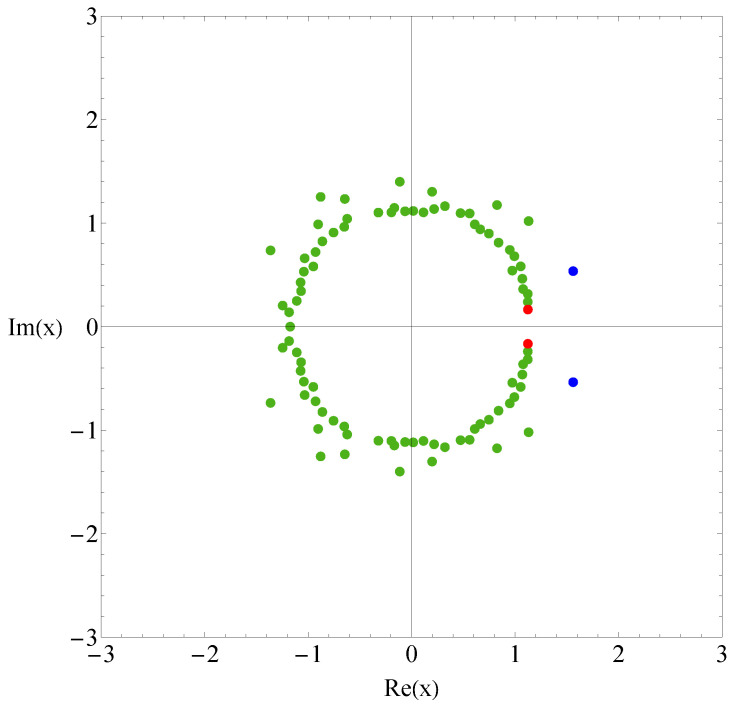
Partition function zeros for sequence A. Red and blue dots indicate the first zeros associated with the collapse and folding transitions, respectively.

**Figure 2:**
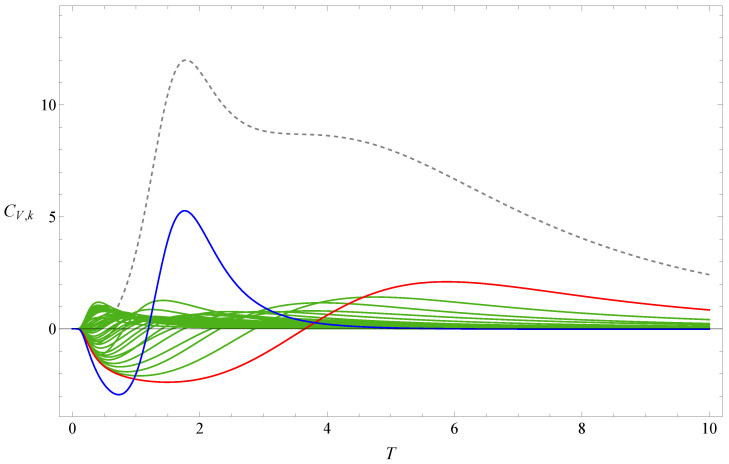
Heat capacity component $C_{V,\;k}$ of sequence A. The dashed line represents the heat capacity $C_{V}$. The red and blue lines, respectively, represent the heat-capacity components contributed by the red and blue first zeros shown in Figure 1.

Figure 1 shows the the partition function zeros of sequence A, and Figure 2 displays the associated heat capacity components $C_{V,\;k}$ in the same colors. Note that in the two figures, the variables *x* and *T* are inversely related: T=1/log(x), where $k_{B}$ is set to 1 by adjusting the temperature unit. The locus of the partition function zeros reveals a clear inner ring corresponding to the collapse transition and a diffuse outer ring corresponding to the folding transition. The first zeros are marked in red and blue, respectively. Since two complex-conjugate zeros contribute the same heat capacity component, we plot $2\;C_{V,\;k}$ for the pair of $x_{k}$ and its complex conjugate, except in very few cases where the zeros lie on the negative real axis; in those cases, only the heat capacity component contributed by one zero is shown. Individual heat capacity components may take negative values. However, when summed, these negative parts are cancelled out by other positive parts, resulting in an entirely positive total heat capacity. The peak regions of the components contribute most to the total heat capacity, and these are, of course, always positive.

**Figure 3:**
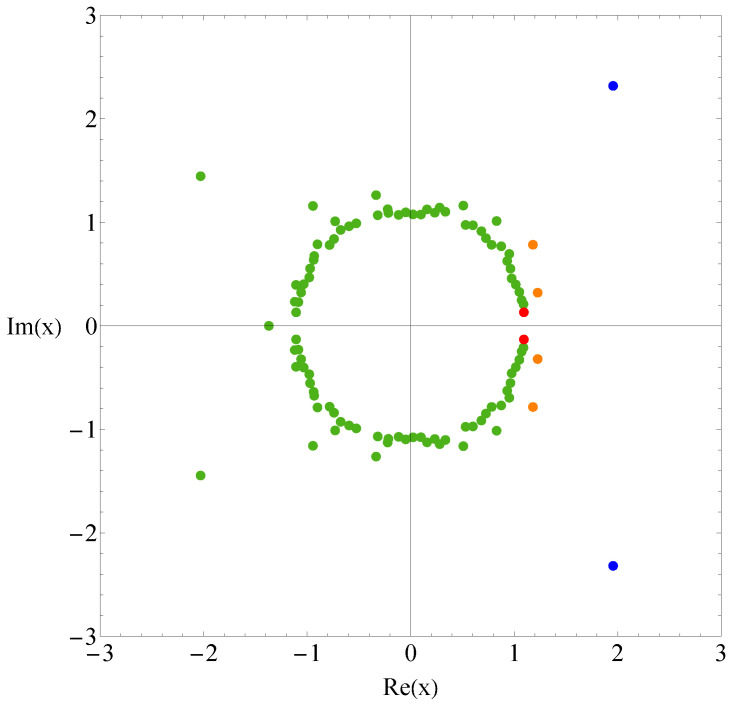
Partition function zeros for sequence B. Red and blue dots indicate the first zeros associated with the collapse and folding transitions, while there are intermediate orange dots between them.

**Figure 4:**
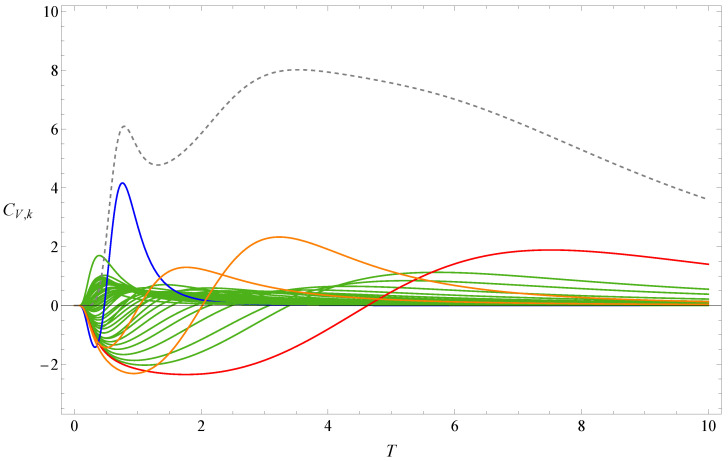
Heat capacity component $C_{V,\;k}$ of sequence B. The dashed line represents the heat capacity $C_{V}$. The red and blue lines, respectively, represent the heat-capacity components contributed by the red and blue first zeros shown in Figure 3.

**Figure 5:**
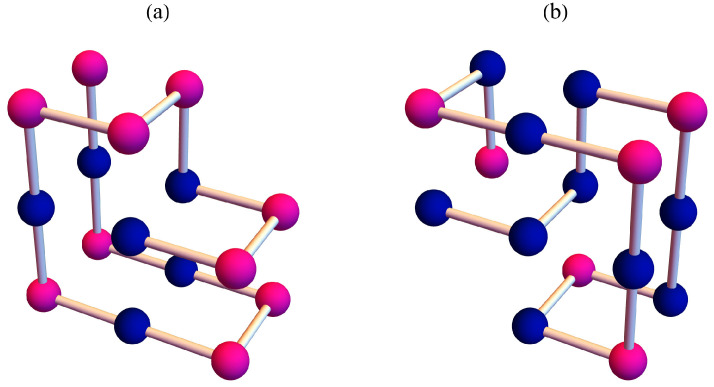
The native states for (**a**) sequence A and (**b**) sequence B. The dark blue beads represent H amino acids, and the pink beads represent P amino acids.

Let us compare the results of sequence B with those of sequence A. Figure 3 and Figure 4 show the the partition function zeros and associated heat capacity components of sequence B. The ring of partition function zeros corresponding to the collapse transition remains evident; however, additional orange zeros appear between the red and blue zeros, such that the locus of zeros resembles three rings rather than two. The orange heat capacity components likewise exhibit significant peaks between the red and blue peaks. A reasonable explanation is that, in this case, more metastable compact globules compete with the native state. As shown in Figure 5, the native state of sequence A is efficiently designed to maximize HH contacts and to greatly reduce the possibility of competing low-energy structures. In contrast, the native state of sequence B is not uniquely favored.The number of structures n(E), listed in the Appendix A, further indicates that sequence B possesses a greater number of low-energy states than sequence A. Since an abundance of metastable states increases the likelihood of kinetic trapping, the folding process is slowed down. Consequently, the folding kinetics of sequence B is expected to be slower than that of sequence A. To verify this prediction, we perform kinetic Monte Carlo simulations for both sequences.

In our Monte Carlo simulations, trial moves are restricted to corner flips and 90-degree end flips, while 180-degree end flips are excluded. Thus, in each Monte Carlo step, only one bead moves, and it is displaced by $\sqrt{2}$ lattice spacing. Other local moves, such as crankshaft moves and pull moves [36], are not included because they involve the movement of multiple beads. We employ this small-step motion to mimic physical movement. Nonlocal moves, such as pivot moves [37] and bond-rebridging moves [38], are considerably more efficient for searching the native state and computing the density of states; however, since they are not physical, they are also excluded from the simulations.

**Figure 6:**
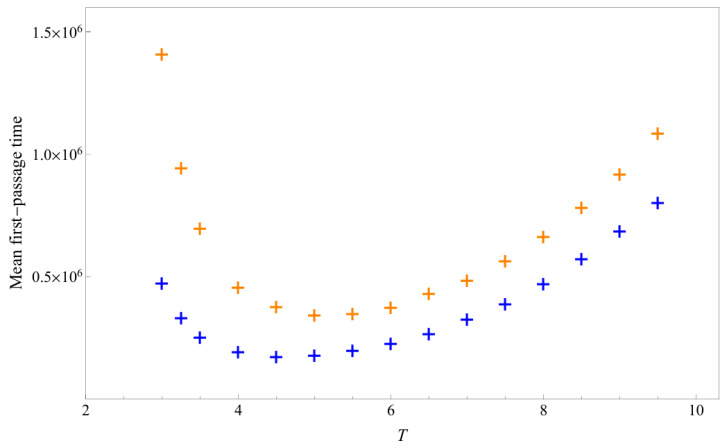
Mean first-passage time of sequence A (blue cross) and sequence B (orange cross).

At each fixed temperature, we conduct 10,000 kinetic Monte Carlo simulations to measure the first-passage time required for a HP sequences to reach its native state from a straight chain. Figure 6 shows the mean first-passage time at various temperatures. Each data point has a relative error of about 1%. The curves exhibit a typical U-shaped profile, a pattern commonly observed in various optimization problems [39]. The underlying reason is that, at high temperatures, entropic effects dominate, reducing the probability of reaching the native state, and at low temperatures, energetic effects dominate, leading to more severe trapping in metastable states.

The minimum of the U-curve indicates the optimal temperature for reaching the ground state. This temperature is approximately 4.5 for sequence A and 5.0 for sequence B. For both sequences, this optimal temperature lies between $T_{\theta }$ and $T_{f}$. The specific values are $T_{\theta }=5.876219$ and $T_{f}=1.763969$ for sequence A, and $T_{\theta }=7.512336$ and $T_{f}=0.758160$ for sequence B. Across all temperatures, sequence A reaches its native state more rapidly than sequence B. This result confirms the prediction based on the analysis of partition function zeros and heat capacity decomposition. At higher and lower temperatures, data with reduced statistical precision also support this conclusion.

Klimov and Thirumalai [40] proposed a criterion for the foldability of proteins: a sequence is considered an efficient folder if $|T_{\theta }-T_{f}|/T_{\theta }$ is small. The present results are consistent with this criterion. Furthermore, heat capacity decomposition provides additional insight into the critical region between $T_{\theta }$ and $T_{f}$. When the collapse and folding transitions are separated by intermediate crossovers induced by metastable compact globules, the folding speed of an HP chain may be reduced. This scenario is exemplified by sequence B. Being grounded in thermodynamic properties, this new criterion exhibits a high degree of generality and applicability to sequences across different models.

## 4. Discussion

Heat capacity decomposition based on partition function zeros is an entirely new approach that focuses directly on thermodynamic properties rather than relying on statistical perspectives. It provides an X-ray–like view of the heat capacity and offers new insight into the sequence’s folding behavior. In this paper, we apply heat capacity decomposition to the HP model for simplicity; however, this approach is general and easily extendable to other model types, including off-lattice or all-atom models. For instance, Alves and Hansmann [41] demonstrated the applicability of the partition function zeros approach to systems using the standard all-atom ECEPP/2 force field. By discretizing the energy into intervals of equal lengths, the partition function remains a polynomial. Therefore, the partition function zeros are readily calculable, permitting the application of the heat capacity decomposition method.

Our ongoing follow-up work focuses on two primary tasks: the design of a folding network to explore metastable states and the computation of partition functions for longer chains. In addition to sequences A and B, we have analyzed the partition function zeros of all the remaining 5246 HP sequences. The inner circle of zeros associated with the collapse transition exhibits a high degree of similarity, suggesting that this transition is a universal characteristic of polymers and is not dependent on the specific sequence. In contrast, the zero distribution corresponding to the folding transition is quite divergent, as illustrated by sequences A and B. Its pattern primarily depends on the number of metastable states that the sequence can form. Metastable states compete with the native state, leading not only to kinetic trapping but also to a weakened folding transition. Exploring these metastable states requires a depiction of the energy landscape, which is a technically challenging task. The kinetic Monte Carlo simulations employed in this study can be used to map the energy landscape. The state space can be represented by a folding network, in which only low-energy states serve as nodes. The weighted connections between these nodes are obtained from Monte Carlo trajectories and can be converted into distance metrics. Combining this two-dimensional folding network with one-dimensional energy yields a simplified three-dimensional energy landscape. The folding properties of a sequence can be inferred from whether its energy landscape is funnel-like or glassy. The folding network is currently under construction.

The most time-consuming step of the entire calculation is computing the partition function. We employ exact enumeration, which can generate partition functions for longer chains when not all sequences are considered. In the future work, we plan to select several 27-residue HP sequences with well-designed native structures that fit into a 3 × 3 × 3 cube and compute their partition functions. Longer chains are advantageous because they produce a denser and smoother locus of partition function zeros, thereby enabling more quantitative analyses. For even longer chains, approximation methods are necessary. Wang–Landau sampling [42] is a type of multicanonical algorithm in which the density of states is determined by adaptively updating to achieve flat-histogram sampling of energies. Wang-Landau simulation has been shown to be a practical and effective method to obtain the partition functions in the HP model [26,43]. To ensure comprehensive sampling of the conformational space, this simulation will incorporate a complete set of efficient moves, including pull, pivot, and bond-rebridging moves.

In summary, we use heat capacity decomposition based on partition function zeros to investigate the foldability of HP sequences. This approach provides a novel perspective not available in previous methods. It can be directly applied to various other protein folding models and is a promising approach for future research.

## Figures and Tables

**Table 1:** Ground-state energy ($E_{0}$), folding temperature ($T_{f}$), and collapse temperature ($T_{\theta }$) of 20 sample HP sequences.

No.	HP Sequence	# of H	$E_{0}$	$T_{f}$	$T_{\theta }$
1	PHPPPPHPPHPHPPPP	4	−72	0.811420	5.140083
2	HPPPHPPPPPPHHPHP	5	−78	0.861228	5.275736
3	PHPPPHPPPPPPHPHH	5	−78	0.631339	5.341242
4	PHPHHPPHHHPPPPPP	6	−81	0.632632	6.053996
5	PHPHHPHPPHPPPPHP	6	−87	1.646424	5.776825
6	HPPHPPPHPHPPHPHP	6	−91	1.763969	5.876219
7	PHHHPPPHPPHHPPHP	7	−90	1.193511	6.236811
8	PHPHHPPHPPPHHPHP	7	−90	1.471768	6.260434
9	HPPPPHPPPHHPHPHH	7	−93	0.235701	6.140142
10	HHHPPHPPPPHPHHPP	7	−93	1.069987	6.278046
11	HHHPHPPPPHPPHHPP	7	−93	1.790133	6.379248
12	HHHPHHPHHPPPHPPP	8	−95	0.788043	6.769531
13	HHPPHHPPHHHPPPHP	8	−96	0.810405	6.598124
14	HHHHPHPHPPHPPHPP	8	−97	1.226218	6.722472
15	HHHPPHPPPHPHPPHH	8	−100	0.943834	6.640664
16	HPHHPHHHPPHPHPHP	9	−100	0.820632	7.186494
17	HHHPHHPPHHHPPHPP	9	−103	1.008023	7.196576
18	HHHHPHHPHPHPHPHP	10	−103	0.758160	7.512336
19	HPHHHHPHHPHPHPHP	10	−103	0.725455	7.638136
20	HHHPHHPHHPPPHPPP	11	−112	0.236975	7.923537

## Data Availability

The original contributions presented in this study are included in the article. Further inquiries can be directed to the corresponding author.

## Appendix A

The partition functions of sequence A, $Z_{16,\;A}\left(x\right)$, and sequence B, $Z_{16,\;B}\left(x\right)$. The coefficient represents the number of structures whose energy corresponds to the negative power of *x*. The sum of all coeffcients, i.e., the total number of structures, is 3,529,191,009.

$$\begin{array}{cc} Z_{16,\;A}\left(x\right)= & \;8x^{91}+56x^{87}+8x^{86}+40x^{85}+40x^{84}+248x^{83}+96x^{82}+664x^{81}+736x^{80}+1024x^{79}+1944x^{78}\\ + & \;3736x^{77}+4560x^{76}+5200x^{75}+10,080x^{74}+12,344x^{73}+13,008x^{72}+23,928x^{71}+35,128x^{70}+33,888x^{69}\\ + & \;39,352x^{68}+81,368x^{67}+82,048x^{66}+76,336x^{65}+132,904x^{64}+206,760x^{63}+152,700x^{62}+251,360x^{61}\\ + & \;384,576x^{60}+316,256x^{59}+399,808x^{58}+723,964x^{57}+640,180x^{56}+647,616x^{55}+1,240,000x^{54}+1,267,140x^{53}\\ + & \;975,540x^{52}+1,917,680x^{51}+2,440,008x^{50}+1,586,320x^{49}+2,668,748x^{48}+4,207,876x^{47}+2,916,452x^{46}\\ + & \;3,820,764x^{45}+6,817,176x^{44}+5,043,796x^{43}+4,741,788x^{42}+10,804,780x^{41}+8,895,120x^{40}+6,272,560x^{39}\\ + & \;15,136,020x^{38}+15,330,928x^{37}+8,273,284x^{36}+21,402,832x^{35}+24,640,816x^{34}+10,663,308x^{33}+26,793,456x^{32}\\ + & \;40,662,032x^{31}+15,906,556x^{30}+30,492,408x^{29}+60,314,884x^{28}+22,970,116x^{27}+33,726,456x^{26}+88,087,996x^{25}\\ + & \;38,991,676x^{24}+20,885,136x^{23}+118,932,704x^{22}+72,575,164x^{21}+22,474,844x^{20}+139,616,276x^{19}\\ + & \;111,308,852x^{18}+183,540,596x^{16}+179,179,144x^{15}+98,424,164x^{13}+260,545,756x^{12}+148,928,892x^{10}\\ + & \;336,550,072x^{9}+491,621,640x^{6}+306,341,992x^{3}+514,975,297 \end{array}$$

$$\begin{array}{cc} Z_{16,\;B}\left(x\right)= & \;8x^{103}+104x^{100}+480x^{99}+200x^{97}+488x^{96}+2488x^{95}+856x^{94}+1688x^{93}+3656x^{92}+4408x^{91}\\ + & \;6360x^{90}+5144x^{89}+8608x^{88}+11,800x^{87}+18,752x^{86}+8736x^{85}+29,392x^{84}+44,840x^{83}+54,560x^{82}\\ + & \;37,088x^{81}+118,112x^{80}+85,568x^{79}+119,880x^{78}+135,960x^{77}+281,496x^{76}+127,992x^{75}+344,056x^{74}\\ + & \;252,400x^{73}+336,600x^{72}+324,480x^{71}+817,884x^{70}+285,736x^{69}+794,744x^{68}+756,708x^{67}+1,142,548x^{66}\\ + & \;602,912x^{65}+2,224,144x^{64}+947,360x^{63}+1,662,784x^{62}+1,524,312x^{61}+3,621,004x^{60}+1,059,240x^{59}\\ + & \;4,268,860x^{58}+2,531,556x^{57}+3,539,792x^{56}+2,490,748x^{55}+8,947,208x^{54}+2,441,064x^{53}+6,948,424x^{52}\\ + & \;5,170,152x^{51}+9,581,796x^{50}+3,539,576x^{49}+16,992,084x^{48}+6,295,624x^{47}+10,576,864x^{46}+7,953,384x^{45}\\ + & \;23,982,100x^{44}+5,387,768x^{43}+27,334,844x^{42}+12,681,076x^{41}+17,141,844x^{40}+9,685,184x^{39}+51,569,408x^{38}\\ + & \;12,338,404x^{37}+35,499,840x^{36}+20,102,820x^{35}+44,555,252x^{34}+8,366,248x^{33}+89,241,520x^{32}+25,271,100x^{31}\\ + & \;33,803,716x^{30}+24,559,676x^{29}+105,881,932x^{28}+12,158,944x^{27}+121,521,252x^{26}+41,509,852x^{25}\\ + & \;52,816,056x^{24}+18,504,412x^{23}+198,963,972x^{22}+25,344,328x^{21}+96,854,212x^{20}+52,563,824x^{19}\\ + & \;144,607,532x^{18}+294,779,568x^{16}+47,636,060x^{15}+37,106,240x^{13}+306,395,492x^{12}+263,352,588x^{10}\\ + & \;68,380,244x^{9}+516,788,024x^{6}+58,447,672x^{3}+514,975,297 \end{array}$$

## References

[1] Anfinson C.B. (1973). Principles that govern the folding of protein chains. Science.

[2] Levinthal C. (1968). Are there pathways for protein folding?. Ext. J. Chim. Phys..

[3] Leopold P.E., Montal M., Onuchic J.N. (1992). Protein folding funnels: A kinetic approach to the sequence-structure relationship. Proc. Natl. Acad. Sci. USA.

[4] Bryngelson D., Onuchic J.N., Socci N.D., Wolynes P.G. (1995). Funnels, pathways, and the energy landscape of protein folding: A synthesis. Proteins.

[5] Dill K.A., Chan H.S. (1997). From Levinthal to pathways to funnels. Nat. Struc. Bio..

[6] Onuchic J.N., Luthey-Schulten Z., Wolynes P.G. (1997). Theory of protein folding: The energy landscape perspective. Annu. Rev. Phys. Chem..

[7] Dobson C.M. (2003). Protein folding and misfolding. Nature.

[8] Dill K.A., MacCallum J.L. (2012). The protein-folding problem, 50 years on. Science.

[9] Nassar R., Dignon G.L., Razban R.M., Dill K.A. (2021). The Protein folding problem: The role of theory. J. Mol. Bio..

[10] Jumper J., Evans R., Pritzel A., Green T., Figurnov M., Ronneberger O., Tunyasuvunakool K., Bates R., Žídek A., Potapenko A. (2021). Highly accurate protein structure prediction with AlphaFold. Nature.

[11] Varadi M., Bertoni D., Magana P., Paramval U., Pidruchna I., Radhakrishnan M., Tsenkov M., Nair S., Mirdita M., Yeo J. (2024). AlphaFold Protein Structure Database in 2024: Providing structure coverage for over 214 million protein sequences. Nucleic Acids Res..

[12] Abramson J., Adler J., Dunger J., Evans R., Green T., Pritzel A., Ronneberger O., Willmore L., Ballard A.J., Bambrick J. (2024). Accurate structure prediction of biomolecular interactions with AlphaFold 3. Nature.

[13] Yang C.N., Lee T.D. (1952). Statistical theory of equations of state and phase transitions. I. Theory of condensation. Phys. Rev..

[14] Lee T.D., Yang C.N. (1952). Statistical theory of equations of state and phase transitions. II. Lattice gas and Ising model. Phys. Rev..

[15] Borrmann P., Mulken O., Harting J. (2000). Classification of phase transitions in small systems. Phys. Rev. Lett..

[16] Janke W., Kenna R. (2001). The strength of first and second order phase. J. Stat. Phys..

[17] Janke W., Johnston D.A., Kenna R. (2004). Phase transition strength through densities of general distributions of zeroes. Nucl. Phys. B.

[18] Lau K.F., Dill K.A. (1989). A lattice statistical mechanics model of the conformational and sequence spaces of proteins. Macromolecules.

[19] Chen C.N., Lin C.Y. (2005). Partition function zeros of the two-dimensional HP model for protein folding. Physica A.

[20] Lee J.H., Kim S.Y., Lee J. (2015). Study on collapse and folding transitions of a lattice protein using exact enumeration. AIP Adv..

[21] Chen C.N., Hsieh Y.H., Hu C.K. (2013). Heat capacity decomposition by partition function zeros for interacting self-avoiding walks. Europhys. Lett..

[22] Dill K.A. (1990). Dominant forces in protein folding. Biochemistry.

[23] Li H., Tang C., Wingreen N.S. (1997). Nature of driving force for protein folding: A result from analyzing the statistical potential. Phys. Rev. Lett..

[24] Chen H., Zhow X., Ou-Yang Z.C. (2001). Difference between proteinlike and nonproteinlike heteropolymers. Phys. Rev. E.

[25] Berger B., Leighton T. Protein folding in the HP model is NP-complete. Proceedings of the Second Annual International Conference on Computational Molecular Biology.

[26] Wüst T., Landau D.P. (2009). Versatile approach to access the low temperature thermodynamics of lattice polymers and proteins. Phys. Rev. Lett..

[27] Fisher M.E. (1965). The nature of critical points. Lectures in Theoretical Physics.

[28] Gross D.H.E., Votyakov E.V. (2000). Phase transitions in small systems. Eur. Phys. J. B..

[29] Dunkel J., Hilbert S. (2006). Phase transitions in small systems Microcanonical vs. canonical ensembles. Physica A.

[30] Vogel T., Bachmann M., Janke W. (2007). Freezing and collapse of flexible polymers on regular lattices in three dimensions. Phys. Rev. E.

[31] Gillespie D.T. (1976). A general method for numerically simulating the stochastic time evolution of coupled chemical reactions. J. Comput. Phys..

[32] Bortz A.B., Kalos M.H., Lebowitz J.L. (1975). A new algorithm for Monte Carlo simulation of Ising spin systems. J. Comput. Phys..

[33] Dinner A.R., Abkevich V., Shakhnovich E., Karplus M. (1999). Factors That Affect the Folding Ability of Proteins. Proteins.

[34] Hsieh Y.H., Chen C.N., Hu C.K. (2016). Efficient algorithm for computing exact partition functions of lattice polymer models. Comput. Phys. Commun..

[35] Huang S.S., Hsieh Y.H., Chen C.N. (2022). Exact enumeration approach to estimate the theta temperature of interacting self-avoiding walks on the simple cubic lattice. Polymers.

[36] Lesh N., Mitzenmacher M., Whitesides S. A complete and effective move set for simplified protein folding. Proceedings of the 7th Annual International Conference on Research in Computational Molecular Biology.

[37] Madras N., Sokal A.D. (1988). The pivot algorithm A highly efficient Monte Carlo method for the self-avoiding walk. J. Stat. Phys..

[38] Deutsch J.M. (1997). Long range moves for high density polymer simulations. J. Chem. Phys..

[39] Chen C.N., Chou C.I., Hwang C.R., Kang J., Lee T.K., Li S.P. (1999). Monte Carlo dynamics in global optimization. Phys. Rev. E.

[40] Klimov D., Thirumalai D. (1996). Criterion that determines the foldability of proteins. Phys. Rev. Lett..

[41] Alves N.A., Hansmann U.H.E. (2000). Partition Function Zeros and Finite Size Scaling of Helix-Coil Transitions in a Polypeptide. Phys. Rev. Lett..

[42] Wang F., Landau D.P. (2001). Efficient, Multiple-Range Random Walk Algorithm to Calculate the Density of States. Phys. Rev. Lett..

[43] Farris A.C.K., Landau D.P. (2021). Replica exchange Wang–Landau sampling of long HP model sequences. Physica A.

